# Possible renoprotective mechanisms of SGLT2 inhibitors

**DOI:** 10.3389/fmed.2023.1115413

**Published:** 2023-03-09

**Authors:** Akira Nishiyama, Kento Kitada

**Affiliations:** Department of Pharmacology, Faculty of Medicine, Kagawa University, Takamatsu, Japan

**Keywords:** SGLT2 inhibitor, chronic kidney disease (CKD), diabetic kidney disease (DKD), renal function, metabolism, aestivation-like response

## Abstract

Treatment with a sodium glucose cotransporter 2 (SGLT2) inhibitor in patients with chronic kidney disease reduces the renal risk independent of changes in blood glucose concentrations and blood pressure. However, the precise mechanism responsible for this SGLT2 inhibitor-induced renoprotective effect is unclear. We have previously shown that SGLT2 inhibitors induce antihypertensive effects with decreased sympathetic nerve activity, which is associated with transient natriuresis. Furthermore, treatment with an SGLT2 inhibitor improves renal ischemia by producing vascular endothelial growth factor-a in the renal tubules. Other studies have suggested that ketone body production, changes in glomerular hemodynamics, and intrarenal metabolic changes and a reduction in oxidative stress due to decreased tubulointerstitial glucose levels may also be involved in the renoprotective effects of SGLT2 inhibitors. In this review, we summarize the mechanism responsible for the SGLT2 inhibitor-induced renoprotective effects, including our recent hypothesis regarding an “aestivation-like response,” which is a biological defense response to starvation.

## 1. Introduction

Sodium glucose cotransporter 2 (SGLT2) is primary expressed in the S1 and S2 segments of the proximal tubules. Therefore, SGLT2 inhibitors suppress glucose reabsorption at the proximal tubules and induce glucosuria. As a result, a reduction in blood sugar concentrations is accompanied by caloric loss (1, 2). However, recent large-scale, clinical trials have shown that treatment with SGLT2 inhibitors have improved renal outcome not only in patients with diabetic kidney disease (DKD), but also in patients with chronic kidney disease (CKD) without diabetes (3–5). Although complex factors may be involved in the SGLT2 inhibitor-induced renoprotective effects, the precise mechanism involved is unclear.

SGLT2 is a glucose transporter that reabsorbs glucose and sodium ions together in a 1:1 ratio in the S1 and S2 segments of the proximal tubule (1). Therefore, SGLT2 inhibitors should block the reabsorption of glucose and sodium ions and increase their concentration in the tubular lumen at this site. However, other glucose transporters and sodium channels are located at distal sites, which may buffer the effects of an SGLT2 inhibitor to some extent. Approximately 90% of the glucose filtered by the glomerulus is thought to be reabsorbed by SGLT2 expressed at the S1 and S2 segments of the proximal tubule, whereas the remaining 10% is reabsorbed by SGLT1 expressed at the distal S3 segment of the proximal tubule (1). Therefore, even when SGLT2 is completely inhibited by SGLT2 inhibitors, a certain amount of increased glucose in the tubular lumen due to inhibited reabsorption is reabsorbed by SGLT1 expressed in the more distal segment. SGLT1 has a high reserve capacity for glucose reabsorption and can reabsorb one glucose and two sodium ions together (1). Therefore, SGLT1 is assumed to reabsorb large amounts of sodium because of its high glucose reabsorption reserve capacity. Consequently, although an SGLT2 inhibitor is a simple agent that inhibits SGLT2 in the proximal tubular S1 and S2 segments, it may considerably alter the environment within the kidney. In this review article, we briefly discuss our hypotheses regarding the possible mechanism of SGLT2 inhibitor-induced renoprotective effects.

## 2. SGLT2 inhibitor-induced systemic changes and their renoprotective effects

### 2.1. Hypoglycemic effect

SGLT2 inhibitors were developed as hypoglycemic agents to control blood glucose concentrations in patients with diabetes. In patients with DKD, lowering blood glucose concentrations elicits a beneficial effect on renal injury. Several studies have shown that high glucose concentrations cause oxidative stress in the kidney, which induces renal tissue injury (6). Obesity also causes metabolic abnormalities in the kidney, which may be ameliorated by caloric loss with an SGLT2 inhibitor. Tanaka et al. (7) showed that the administration of an SGLT2 inhibitor or dietary restriction improves kidney tissue metabolism and oxidative stress in obese type 2 diabetic mice.

Ketone bodies are produced in the body as energy when blood glucose concentrations are lowered owing to caloric loss (8). Tomita et al. (9) showed that ketone bodies produced by SGLT2 inhibitor administration led to a protective effect on glomerular and tubular injury in obese diabetic mice. However, caloric loss promotes gluconeogenesis in the kidney. To the best of our knowledge, a specific effect of gluconeogenesis on renal injury has not yet been clarified.

### 2.2. Blood pressure reduction

In hypertensive patients with CKD, adequate blood pressure control is important to attenuate the progression of renal dysfunction (10). SGLT2 inhibitors have been clinically shown to have an antihypertensive effect (11). We have also reported that, in the Otsuka Long Evans Tokushima Fatty rat (animal model of obesity) and in the SHR/NDmcr-cp animal model of metabolic syndrome, treatment of an SGLT2 inhibitor normalized the dipping pattern of blood pressure in addition to its antihypertensive effects (12, 13). The pharmacological mechanism for the antihypertensive effect of SGLT2 inhibitors may be through weight loss and hypoglycemia. However, most of the antihypertensive effects of SGLT2 inhibitors do not correlate with changes in body weight or blood glucose concentrations (11). Another possible mechanism is the improvement of insulin resistance by SGLT2 inhibitors. Clinical studies have reported that hyperinsulinemia may contribute to high blood pressure by sodium increasing sodium reabsorption in the kidney (14). However, in the above-mentioned animal studies with Otsuka Long Evans Tokushima Fatty rats and the SHR/NDmcr-cp model, an improvement in diurnal blood pressure variability by SGLT2 inhibitors was accompanied by increased urinary sodium excretion (12, 13, 15). These data suggest that the natriuretic effect of SGLT2 inhibitors is also involved in lowering blood pressure. In fact, our clearance studies in rats showed that acute administration of an SGLT2 inhibitor induced a transient increase in urinary sodium ion excretion, which was accompanied by an increase in urinary glucose concentrations (16).

### 2.3. Aestivation-like response

SGLT2 inhibitors suppress the reabsorption of glucose and sodium at the proximal tubule, and urinary glucose becomes an osmolyte in the urine and induces osmotic diuresis (1). These effects of SGLT2 inhibitors should be associated with glucose loss and fluid loss. With regard to glucose loss due to SGLT2 inhibitors, the hypoglycemic effect is likely to continue because of the sustained increase in urinary glucose concentrations during treatment. However, in response to such caloric loss, hunger is experienced, and energy is achieved through internal ketone body production and glucogenesis (8). Therefore, many clinical reports have shown that weight loss does not continue for a long period of time and is limited to a loss of 2–3 kg in a few months (3–5). However, a reduction in body fluid induced by an SGLT2 inhibitor is accompanied by osmotic diuresis, which lasts only a few days (17). Immediately after SGLT2 inhibitor administration, diuresis (i.e., fluid loss) with increased urine volume and urinary sodium excretion occurs for several days, but this diuretic effect usually diminishes soon thereafter owing to the adaptive activation of the renin–angiotensin and vasopressin systems (18). Therefore, although SGLT2 inhibitors cause caloric and fluid loss, various compensatory mechanisms in the body are triggered to counteract these losses. As a result, the organism is maintained in a constant state, which is thought to minimize the appearance of serious side effects of an SGLT2 inhibitor.

Among the various compensatory mechanisms that occur *in vivo* upon SGLT2 inhibitor administration, we have proposed the possibility of an “aestivation-like response” as an adaptive response (19, 20). Aestivation is observed in lungfish and amphibians, and it retains body fluids by producing osmotic substances such as urea (21). We recently found that part of this process is also maintained in humans, which we named the “aestivation-like response” (21). Interestingly, metabolomic analysis of metabolic changes in patients with type 2 diabetes has shown that treatment with an SGLT2 inhibitor promotes a catabolic state, which is involved in the urea cycle and branched differentiated amino acids (22). Our data have also shown that, during an aestivation-like response, energy shifts to the urea cycle and other processes occur, and sympathetic nerves are inhibited accordingly (23). Similarly, several studies have shown that SGLT2 inhibitor administration suppresses sympathetic nerve activity (24, 25). These findings suggest that caloric and body fluid losses due to SGLT2 inhibitor administration may induce metabolic energy and water conservation. This conservation is associated with an aestivation-like response, such as protein catabolism and the suppression of sympathetic nerve activity (19, 20, 23). We speculate that the body’s latent biological defense capacity is activated by an SGLT2 inhibitor, which may be partly responsible for organ protection. Obviously, our hypothesis is still speculative and requires further validation. The differences between “aestivation” and “aestivation-like response” are briefly summarized in Table 1.

**TABLE 1:** Similarities and differences between aestivation and aestivation-like response for water conservation.

	Aestivation	Aestivation-like response
Fasting	Yes	No
Water deprivation	Yes	No
Dormancy	Yes	No
Metabolic change for water conservation (ex: osmolyte production)	Yes	Yes
Water conservation in biological barriers (ex: skin, kidney)	Yes	Yes
Osmolyte accumulation	Yes	Yes

## 3. SGLT2 inhibitor-induced changes in renal hemodynamics and their renoprotective effects

### 3.1. Changes in glomerular hemodynamics

Large clinical trials have indicated that an improvement in renal outcome with SGLT2 inhibitors is accompanied by a reduction in the glomerular filtration rate (GFR) and albuminuria (3–5). These data suggest that a reduction in intraglomerular pressure plays an important role in the renoprotective effect of SGLT2 inhibitors. Several investigators have suggested that a reduction in intraglomerular pressure by SGLT2 inhibitors is induced by the activation of a tubuloglomerular glomerular feedback (TGF) mechanism (2, 26). When SGLT2 inhibitors are administered, a large amount of solutes, such as glucose and sodium ions, reach the macula densa at the distal tubules where TGF mechanism is activated (27). Macula densa cells sense an increase in solutes in the tubular lumen and release ATP and adenosine extracellularly. This release causes the nearby glomerular afferent arterioles to constrict, leading to a reduction in intraglomerular pressure (28). Therefore, SGLT2 inhibitors may reduce intraglomerular pressure (simultaneously lowering GFR), thereby improving glomerular hyperfiltration and protecting the kidney. However, no reports have shown a positive relationship between the incidence of renal events and a reduction in the GFR or albuminuria. In addition, animal studies in both mice and rats have shown inconsistent data on the effects of SGLT2 inhibitors on the GFR and albuminuria (15, 16). Another study reported that SGLT2 inhibitors affect mesangial cellular contraction, which is possibly involved in the regulation of the GFR by the TGF mechanism (29).

Recently, Hare et al. (30) showed that, in a rat model of type 1 diabetes, SGLT2 inhibitor treatment decreased tissue oxygen saturation in the deep renal cortex and outer medulla. This decrease was associated with a decreased GFR and increased blood erythropoietin expression and erythrocyte reticulocyte count. In contrast, no such changes were observed in other studies (31, 32). Therefore, these data suggest that the effects of SGLT2 inhibitors in promoting erythropoietin production in the kidney differ depending on the pathogenesis of CKD.

### 3.2. Other hemodynamic changes

SGLT2 inhibitors increase urinary excretion of glucose and cause osmotic diuresis because glucose is an osmolyte. Water is thought to move from the renal interstitium into the urine, which is associated with a reduction in the amount of renal interstitial water. These effects of SGLT2 inhibitors have been suggested to improve renal congestion (33, 34). Preliminary studies by Hirose et al. (34) showed that treatment with SGLT2 inhibitors improved renal congestion in a model of renal venous coarctation. We also showed that, in a mouse model of renal fibrosis after ischemia–reperfusion, an SGLT2 inhibitor markedly improved renal congestion *via* increased tubular vascular endothelial growth factor-a expression (35). Therefore, SGLT2 inhibitors may improve hemodynamics in perivascular vessels and in the glomeruli. More detailed studies on this possibility are expected in the future.

## 4. SGLT2 inhibitor-induced reduction in renal tubulointerstitial glucose concentrations and its renoprotective effects

As mentioned above, SGLT2 is expressed primarily in the S1 and S2 segments of the proximal tubules, which reabsorb glucose and sodium in a 1:1 ratio. Therefore, SGLT2 inhibitors may reduce glucose concentrations in the tubular cells and interstitial space in this area. However, a detailed study showed that, in normal mice, even when glucose reabsorption was inhibited by an SGLT2 inhibitor, glucose concentrations in the S1 segment proximal tubular cells remained relatively constant because glucose flowed in from the interstitial side *via* glucose transporter type 2 (GLUT2) (35, 36). Interestingly, this compensatory response does not work well in damaged kidneys because GLUT2 expression and function are impaired. As a result, glucose concentrations in proximal tubular cells at the S1 and S2 segments are considerably reduced by treatment with an SGLT2 inhibitor (35). Therefore, in the pathogenesis of CKD, an SGLT2 inhibitor may reduce glucose concentrations in proximal tubular cells and the interstitial space, at least near the S1 and S2 segments, which elicit various effects on tubulointerstitial function. A growing body of experimental evidence has shown that, in the proximal tubular S1 and S2 segments, SGLT2 inhibitors suppress glucose-induced tubular cell epithelial-mesenchymal transition (37), oxidative stress (6, 7), and aging (38). On the other hand, in the distal proximal tubular S3 segment, where SGLT2 is not expressed but SGLT1 is abundant instead (1), SGLT2 inhibitors may increase intracellular glucose concentrations. However, these effects of SGLT2 inhibitors have not been studied in detail.

The effects of an SGLT2 inhibitor have been studied in animal models of polycystic kidney disease (PKD) in which lesions occur further downstream in the collecting ducts (39, 40). In PKD Han: SPRD rats, the administration of an SGLT2 inhibitor did not change cyst size, while the GFR was increased and albuminuria was attenuated (40). However, in PKD PCK rats, treatment with an SGLT2 inhibitor worsened albuminuria and the cyst size (39). These experimental data suggest that SGLT2 inhibitor administration is not recommended in patients with PKD. Thus, SGLT2 inhibitor administration may greatly alter the intrarenal environment not only at the proximal tubular S1 and S2 segments, but also at the distal nephron, and careful observation is required in the future.

## 5. Discussion

SGLT2 inhibitors have attracted attention as agents that improve the prognosis of CKD. This review outlines the currently proposed mechanisms by which SGLT2 inhibitors elicit renoprotective effects. Further determination of the mechanism responsible for SGLT2 inhibitor-induced renoprotective effects will likely lead to the development of new drugs for patients with CKD. Several SGLT2 inhibitors are currently available, but only a limited number of them have proven renoprotective effects. However, based on their respective drug profiles, it is possible that all SGLT2 inhibitors to have similar renoprotective effects.

## Author contributions

AN: manuscript draft preparation and writing—review and editing. KK: review and editing. Both authors contributed to the article and approved the submitted version.
